# Correlation between fluoride release, surface hardness and diametral tensile strength of restorative glass ionomer cements

**DOI:** 10.4317/jced.61499

**Published:** 2024-05-01

**Authors:** Mariana Pardi, Bruna-Mandrá da Cunha, Heitor-Monteiro-Mundim Cunha, Manoela-Borges e Souza Marques, Kaio-Luca-Gimenes Ribeiro, Carlos-Eduardo-Ferreira Cruz, Carla-Regina Costa, César-Penazzo Lepri, Denise-Tornavoi de Castro

**Affiliations:** 1 Department of Biomaterials, University of Uberaba, Uberaba, MG, Brazil; 2 Department of Chemistry, Institute of Exact, Natural and Educational Sciences, Federal University of Triângulo Mineiro, Uberaba, MG, Brazil

## Abstract

**Background:**

The aim of this study was to determine if there is a correlation between fluoride release, surface hardness, and diametral tensile strength of restorative glass ionomer cements (GICs).

**Material and Methods:**

Conventional (Riva Self Cure) and resin-modified (Riva Light Cure) GICs were used. Thirty-four samples (ø 6 x 3 mm) were prepared for each cement. The kinetics of fluoride release (n=4) was evaluated over 28 days using a fluoride-selective electrode (ISE 4010-C00). The analysis of surface hardness (n=10) was performed using a microhardness tester (Shimadzu HMV-2000, Japan) with a Knoop indenter and a load of 25 gf for 30 seconds. The diametral tensile strength test (n=10) was conducted on a universal testing machine at a speed of 0.75 mm/min. Fluoride release data were analyzed by two-way repeated measures ANOVA and Bonferroni post hoc test, while independent t-test was used for other analyses (α=0.05).

**Results:**

Overall, the groups showed higher fluoride release until day 7 and a progressive decrease until day 28. On day 1 and day 21, Riva Self Cure showed a higher level of release than Riva Light Cure (*p*=0.026). Riva Light Cure showed higher diametral tensile strength (*p*<0.0001) and surface hardness (*p*=0.034) than Riva Self Cure. A negative correlation was found, indicating that higher fluoride release is associated with lower surface hardness and diametral tensile strength.

**Conclusion:**

Fluoride release and mechanical performance are related properties of GICs, and these properties exhibit different values depending on the type of material. Resin-modified GIC release less fluoride but exhibit better mechanical performance compared to conventional GIC.

** Key words:**Diametral Tensile Strength, Fluoride, Glass Ionomer Cement, Surface Hardness.

## Introduction

Dental caries is one of the most common chronic diseases, affecting 60 to 90% of school-aged children and about 36% of the world’s population, according to the World Health Organization (1-3).

Glass ionomer cements (GICs) are the bioactive restorative materials of choice in cases of high levels of caries activity (4). In addition to the chemical adhesion to the tooth structure, the ability to release and recharge fluoride are considered important properties for clinical use (5,6).

GIC’s preventive effect on caries progression has been widely discussed in literature and remains unclear, but evidence suggests that its anti-caries activity is linked to a prolonged release of fluoride in mouth, which is absorbed by saliva and surrounding enamel (5,7). However, some studies have shown a correlation between high levels of fluoride release and poor mechanical properties (8,9).

Conventional GICs combine water-soluble polymeric acids with calcium or strontium-based aluminium silicate glass powder and fluoride (3). Despite numerous advantages, the clinical limitations of these materials are mainly related to weak mechanical properties, low wear resistance, susceptibility to moisture during the initial curing reaction, and short working time (4,10).

To minimize these disadvantages and improve the properties, various changes in the composition of GICs have been proposed by adding components such as metals, bioactive glasses, fluorapatite, or resins (11-14). The development of resin-modified GICs, through the incorporation of hydrophilic resin monomers, has provided a combination of the advantageous properties of composite resins and glass ionomer cements. Thus, physical and mechanical properties have become superior to conventional materials, and fluoride release has been maintained, even if at lower levels (15).

Selecting the ideal restorative material is a significant challenge for clinicians due to the wide variety of dental products available (16,17). Considering the place of GICs in restorative dentistry, particularly from a minimally invasive perspective, these materials require study. Specifically, it is important to understand the relationship between mechanical properties and fluoride release, as both are important for long-term durability.

The aim of this study was to evaluate the fluoride release and mechanical properties of surface hardness and diametral tensile strength of a conventional glass ionomer cement (Riva Self Cure) and a resin-modified cement (Riva Light Cure) and to determine if these properties are correlated. The null hypothesis were: 1) there is no difference between the GICs studied in the amount of fluoride released up to 28 days; 2) there is no difference between the GICs studied in terms of mechanical properties; and 3) there is no correlation between fluoride release and mechanical properties.

## Material and Methods

The glass ionomer cements used in this study were Riva Self Cure and Riva Light Cure ( Table 1).

Thirty-four specimens of each material, measuring ø 6 x 3 mm, were obtained using hemisected circular metal matrices. After manipulating each material on a glass plate according to the manufacturer’s proportions, they were inserted into the matrix, which had been previously isolated with vaseline. For the resin-modified cement, two increments were used, the first of which was light-cured for 20 seconds using the Sdi Radii Cal Led device, before the second increment was placed, which was also light-cured for the same period of time after placing a strip of polyacetate to allow the excess material to flow out. After the polymerization reaction, the specimens were removed from inside the matrix, and finished and polished using #400, #600, #1200, #2000, and #2500 grit sandpaper. Samples were rinsed with distilled water between sanding to remove debris.

For fluoride release analysis, the samples were suspended by a nylon thread in polypropylene tubes containing 4 mL of deionized water. They were then incubated at 37°C. Deionized water was replaced at intervals of 1, 7, 14, 21 and 28 days. After this period, the fluoride concentration released in the water samples was evaluated to obtain the fluoride release profile over time for each type of cement.

To obtain the release profile over time for each type of cement, a fluoride ion-selective electrode (ISE 4010-C00), pre-calibrated from the linear regression curve E(mV) vs. log [F-], was used. Potential measurements were made against an Ag/AgCl reference electrode using a potentiometer. For the determination of the calibration curve, nine standard solutions were prepared by diluting a stock fluoride solution with a concentration of 1000 ppm (ISE 4010-C00). The solutions were prepared in 25 mL flasks, with 2.5 mL of total ionic strength adjustment buffer (TISAB) added to each flask, and stock solution volumes ranging from 10 μL to 5000 μL (5 mL). TISAB consisted of a 1 mol/L sodium chloride (NaCl, CRQ Chemicals) solution and a 1 mol/L acetic acid (CH3CO2H, CRQ Chemicals) solution, the pH of which was adjusted to 5.5 with 1 mol/L sodium hydroxide (NaOH) solution. The flasks were then filled with deionized water. After preparation, these solutions were transferred to polyethylene bottles and stored in a refrigerator throughout the study. All measurements were carried out over three days at room temperature, with a new calibration curve drawn for each day of analysis. Values were expressed in ppm F-. Thus, data on the total fluoride released were recorded for each interval. Each condition to be analyzed was replicated 4 times, with the potential measurement performed in triplicate for each of the replicates, and the result was expressed as the mean and standard deviation.

The samples to be subjected to mechanical tests were immersed in deionized water and stored in an oven for 24 hours after polishing. Surface hardness analysis (n=10) was performed using a microhardness tester (Shimadzu HMV-2000, Japan). Three randomly equidistant measurements were made on each specimen using a Knoop indenter with a load of 25 gf (gram force) for 30 seconds. The indentations were measured by two marks at the corners of the rhombus in an image at 40x magnification, and the length of the major diagonal and consequently the Knoop hardness results were determined by automatic calculation by the instrument software.

The diametral tensile strength (DTS) test (n=10) was performed according to ADA Standard No. 66 on a universal testing machine (EMIC DL-3000 - 30 kN capacity) at a speed of 0.75 mm/min. The samples were positioned with their longitudinal side between the plates of the machine and subjected to compressive loading until rupture. The DTS in megapascals (MPa) was calculated using the equation:

T = 2F/π DL

Where T is the resistance, F is the maximum load applied in Newtons (N), D is the diameter of the specimens in mm and L is the length of the specimen in mm (18).

Fluoride release data were analyzed using a two-way repeated measures ANOVA followed by Bonferroni post hoc test, while independent t-tests (α=0.05) were used for other analyses. Statistical analysis was performed using SPSS version 22.0 software.

## Results

The fluoride ion release profiles of Riva Self Cure and Riva Light Cure in deionized water were recorded at 5 specific intervals over 28 days. The amount of fluoride released was documented in parts per million (ppm).

**Figure 1:**
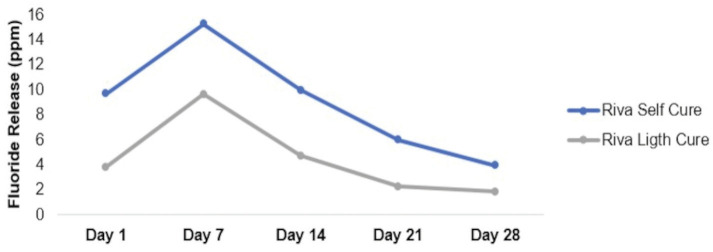
Fluoride release from conventional and modified glass ionomer cements over time.

 Table 2 and Figure 1 show the comparative evaluation of fluoride release considering the time x material interaction.

In general, both cements showed a higher release at the day 7 with a progressive decrease until the day 28 (*p*<0.05). It was observed that Riva Self Cure showed higher fluoride release compared to Riva Light Cure at all time points, with a statistical difference at day 1 and day 21 (*p*=0.026).

**Figure 2:**
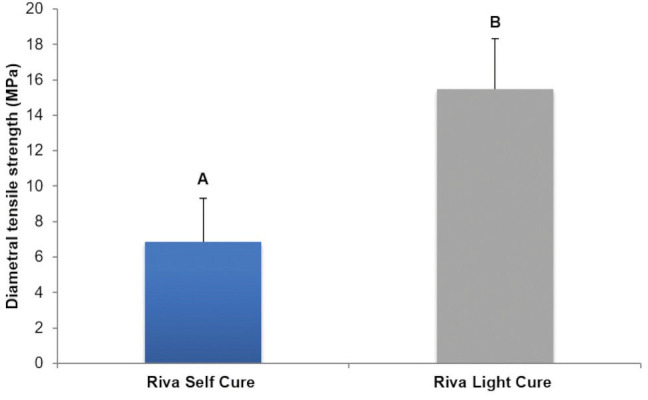
Comparison of diametral tensile strength (MPa) between conventional glass ionomer cement and resin-modified glass ionomer cement.

**Figure 3:**
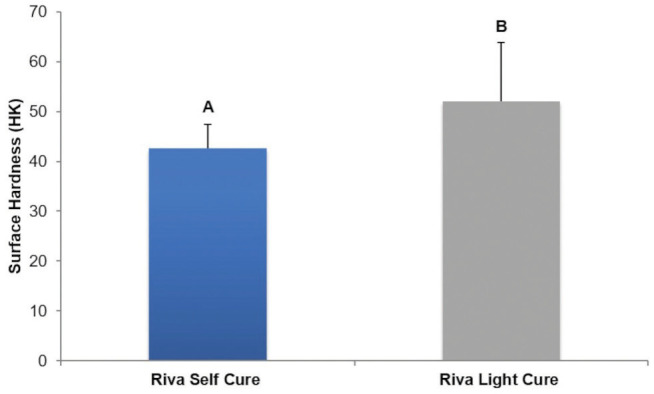
Comparison of surface hardness (HK) between conventional glass ionomer cement and resin-modified glass ionomer cement.

 Riva Light Cure showed higher diametral tensile strength (*p*<0.0001) and surface hardness (*p*=0.034) than Riva Self Cure (Figs. 2,3).

The Pearson correlation coefficient (r) showed a strong negative correlation between fluoride release and diametral tensile strength (r = - 0.892). The correlation between fluoride release and surface hardness was also negative, but moderate (r = -0.468) ( Table 3). The indices considered to indicate the strength of the correlation between variables were: r > 0.70 indicates a strong correlation; 0.30 < r < 0.70 indicates a moderate correlation; r < 0.30 indicates a weak correlation; r ≈ 0 indicates no correlation (19). A positive correlation indicates that an increase in one variable leads to an increase in another, while a negative correlation indicates a decrease in one variable with an increase in another.

## Discussion

This study determined the relationship between fluoride release and the properties of surface hardness and diametral tensile strength of conventional and resin-modified glass ionomer restorative cements.

There is limited data in the literature correlating these variables. The three null hypotheses of the study were rejected because the amount of fluoride released by the cements differed significantly, as did the mechanical properties tested. Furthermore, a correlation between the variables was observed.

Fluoride release from a restorative material is determined by the matrix, the curing mechanism and the amount of fluoride present in the material (8,20,21). Both materials tested showed higher fluoride release within the first 7 cumulative days, tending towards a steady state. This performance is attributed to the greater instability and erosion of glass ionomers during the initial setting period (2). First, there is surface rinsing, which causes a greater initial dissolution of aluminium or phosphate complexes with fluoride formed during the acid-base reaction. This process is mainly controlled by glass particles in the cement that have not reacted. Following the higher fluoride release in the first few days, sustained release can occur during the remaining period by diffusion through pores, fissures and mass diffusion (1).

In general, conventional glass ionomer cements tend to release more fluoride than resin-modified cements, which is consistent with literature data (1). The resin matrix is less hydrophilic, which may contribute to lower fluoride release. Considering that fluoride is released by diffusion through pores and fissures, it can also be speculated that conventional glass ionomer cements have greater matrix porosity, which acts as a pathway for greater fluoride release compared to resin-modified (22).

In the oral cavity, restorative materials, such as glass ionomer cements, are directly exposed to recurrent masticatory and tensile forces (23). Since many clinical failures are due to tensile stress, diametral tensile strength is a critical requirement. The British Standards Institution developed the diametral tensile test because it is impossible to directly determine the tensile strength of brittle materials such as GICs. This test involves compressing a cylindrical specimen around its circumference using compression plates (24).

Conventional GIC is the result of an acid-base reaction between fluoroaluminosilicate powder and polycarboxylic acid. Its adhesion mechanism is based on the formation of a bond between the carboxyl groups of polyacrylic acid and hydroxyapatite on the tooth surface. Studies have reported that these materials are fracture susceptible and have low wear resistance (23). In this study, this group showed lower diametral tensile strength than the resin-modified group, suggesting that they may be less clinically resistant (24).

Hardness testing is also widely used in dentistry as it provides important information about the wear and setting properties of materials (25). In this study, resin-modified GIC showed a higher surface hardness value compared to conventional GIC. These data may indicate a more complete polymerization, as Knoop hardness (KH) has been found to have a strong, significant positive linear correlation with the degree of conversion (26).

Another aspect discussed in the literature is that exposure of GICs to water results in the release of ions, which can lead to changes in the mechanical properties (27). There is a risk of ion loss, which can significantly affect the mechanical properties of the material, if conventional GIC is exposed to water prior to the formation of the calcium aluminium polyacrylate salts. Resin-modified GIC, however, contains hydroxyethyl methacrylate (HEMA), which initiates the curing reaction and prevents the loss of ions (18), showing better mechanical properties.

As shown in  Table 3, there is a negative linear correlation between fluoride release and the mechanical properties evaluated. This indicates that materials with higher fluoride release have lower diametral tensile strength and surface hardness, potentially making them less clinically durable in the load carrying areas.

Therefore, our results clearly showed a negative correlation between fluoride release and mechanical properties, suggesting a link between the two. It is believed that when relatively large amounts of fluoride are transferred from the glass to the matrix during the setting reaction, high fluoride release occurs. The ions are released from the matrix by a combination of early washout and sustained diffusional release. Conventional GICs may be more susceptible to cracking and porosity due to the properties of the matrix itself, as well as having a slower polymerization reaction (acid-base) that favours the transfer of fluoride to the matrix during the setting reaction (4), but they have inferior mechanical performance compared to resin-modified GICs.

The different patterns of fluoride release and mechanical performance of the materials tested may have implications for clinical indications. Further studies are therefore required to determine whether these phenomena are related as suggested.

## Conclusions

Fluoride release and mechanical performance are related properties of GICs and these properties have different values depending on the type of material. Resin-modified glass ionomer cements release less fluoride but have better mechanical performance compared to conventional cements.

## Figures and Tables

**Table 1:** Glass ionomer cements used.

Commercial name/ Manufacturer	Category	Commercial presentation	Main components
Riva self cure^®^/SDI, Bayswater, Victoria, Australia.	Conventional glass ionomer cement	Powder/Liquid	Fluoroaluminosilicate, Polyacrylic acid, Tartaric acid
Riva light cure^®^/SDI, Bayswater, Victoria, Australia.	Glass ionomer cement modified with resin	Powder/Liquid	Fluoroaluminosilicate, Polyacrylic acid, Tartaric acid, Hydroxyethyl methacrylate, Dimethacrylate, Acidified monomer,

**Table 2:** Comparison of fluoride release between conventional glass ionomer cement and resin-modified glass ionomer cement over 28 days (mean ± standard deviation) in ppm.

Groups	Day 1	Day 7	Day 14	Day 21	Day 28
Riva Self Cure	10±1^Aa^	15±1^Ba^	10±3^ABCa^	5.9±0.7^Ca^	4±1^Ca^
Riva Light Cure	4±2^Ab^	9±5^Ba^	5±3^Aa^	2±1^Ab^	2±1^Aa^

**Table 3:** Pearson correlation coefficient (r) between fluoride release, surface hardness, and diametral tensile strength of conventional and resin-modified glass ionomer cements.

Correlation between variables	Pearson coefficient (r)
Fluoride release x Diametral tensile strength	−0.892
Fluoride release x Surface hardness	−0.468

## Data Availability

The datasets used and/or analyzed during the current study are available from the corresponding author.
